# Spectrally Flat and Polarization-Diversified Silicon-Nanowire Coarse WDM Demultiplexers Based on Distributed Multimode-Interference Phase Compensations

**DOI:** 10.3390/nano16161024

**Published:** 2026-08-18

**Authors:** Seok-Hwan Jeong, Heuk Park, Joon Ki Lee

**Affiliations:** 1 Department of Semiconductor Engineering, The University of Suwon, Hwaseong 18323, Republic of Korea; 2 Electronics and Telecommunications Research Institute (ETRI), Daejeon 34129, Republic of Korea; parkh@etri.re.kr (H.P.); juneki@etri.re.kr (J.K.L.)

**Keywords:** silicon-nanowire waveguide, optical filter, optical demultiplexer, polarization control, O-band, coarse wavelength division multiplexing

## Abstract

Spectrally flat-topped and polarization-insensitive coarse wavelength division multiplexing (CWDM)-targeted optical demultiplexers based on distributed multimode-interference (MMI) phase compensations are analytically calculated and experimentally demonstrated. The proposed device for use in a CWDM optical receiver consists of a polarization splitter-rotator (PSR) and two identical silicon-nanowire multiple delayed interferometric (MDI) demultiplexers. The broadband operating nature of MMI couplers is highly suitable for applying the proposed device to >60 nm wide CWDM applications. Moreover, by properly adjusting the relative output phase relations of MMI couplers according to their optical splitting ratios, we experimentally validated a flat-topped CWDM spectral response within the O-band spectral range. Concurrently, stable optical demultiplexing operations were maintained regardless of the input signal polarization states via the monolithically integrated PSR. Fabricated using a silicon photonics foundry process based on ArF-dry lithography technology, the devices exhibited a 1 dB flat bandwidth of >12 nm, a polarization-dependent loss of <1.0 dB, an adjacent-channel isolation of >10 dB, and a polarization crosstalk of <−20 dB across the measured output channels. Although the current spectral crosstalk is ~−10 dB, it can be further suppressed to the −20 dB level through fabrication process optimization to minimize random phase fluctuations, or by incorporating a double-filtering scheme. This distributed MMI phase compensation strategy can be broadly applied to scalable WDM architectures in datacom applications.

## 1. Introduction

Coarse wavelength division multiplexing (CWDM) in the O-band spectral regime has been widely adopted as a cost-effective solution for datacom optical interconnections [1,2,3]. At the optical receiver, wavelength-division-multiplexed (WDM) signals must be spatially separated regardless of their polarization states. To date, optical demultiplexers based on multiple delayed interferometers (MDIs) have demonstrated significant spectral advantages such as compactness, spectral flatness, low out-of-band crosstalk, and low polarization crosstalk, compared to alternative demultiplexing schemes based on microring resonators or arrayed waveguide gratings [4,5,6,7,8,9].

Achieving a flat-topped spectral response fundamentally requires precise control of the power-splitting ratios at each cascaded MDI stage. Prior implementations have frequently utilized directional couplers (DCs) to fulfill this requirement [4,5,6,7,8,9]. Nonetheless, conventional DCs suffer from inherent sinusoidal and wavelength-dependent coupling characteristics, which obstruct the preservation of uniform coupling ratios across the wide CWDM operating band [4,5,7]. While alternative strategies, such as narrow-gap bent DCs (~125 nm) [6,9] or Bezier-shaped DCs [8], have been proposed to mitigate this wavelength dependence, they inevitably compromise the fabrication tolerance within standard silicon photonics foundry platforms [10]. Furthermore, optimizing such complex DC models often demands significant computational resources and high-performance computing (HPC) systems [8,11].

As an alternative to expanding the operational wavelength window, our previous works integrated multimode-interference (MMI) couplers [12,13] into MDI-based CWDM demultiplexers [14,15]. Although the initial spectral profiles were Gaussian-like, we subsequently incorporated asymmetric-coupling-ratio MMI couplers within each MDI stage to synthesize a flat-topped, box-like response, thereby validating the requisite phase dynamics for spectral tailoring [16]. Despite defining the necessary path-length differences and phase adjustments, the previous device fell short of demonstrating a true CWDM-grade spectral response due to uncompensated phase offsets among the three different MMI types. While our recent follow-up work successfully optimized these MMI phase conditions for a long-reach 8 (LR-8) optical demultiplexer [17], its spectral response was not intended for CWDM applications. In addition, the LR-8 spectral behavior was restricted strictly to TE-polarized light, rendering it impractical for optical receivers that must accommodate arbitrary polarization states.

To overcome these limitations, this paper introduces a polarization-diversified architecture integrated with optimized MMI phase engineering to realize a high-performance CWDM demultiplexer featuring flat-topped passbands and low crosstalk. While our recent experimental work employed a double-filtering scheme to suppress the out-of-band crosstalk below −20 dB [18], precise experimental validation of the MMI couplers’ fundamental characteristics remains crucial to improving the low spectral crosstalk property. As indicated in Table 1, in this study, rather than directly extracting individual phase shifts, we accurately verify the specific splitting properties of the MMI couplers by evaluating their integrated amplitude and phase responses through specifically designed MMI-based Mach–Zehnder interferometer (MZI) configurations. Based on these empirical validations, we investigate the fundamental crosstalk limitations (~−10 dB) of standard single-stage MDIs, comprehensively analyzing the realistic impacts of uncompensated phase offsets and practical foundry fabrication tolerances.

## 2. Device Structure and Theoretical Considerations

As discussed in our prior work [16], the relative phase relations of 2:2 MMI couplers are critical for achieving an ideal flat-topped spectral response, because the discrete phase behaviors of MMI couplers, which depend on their optical splitting ratios, directly influence the spectral shape of the MDI-based demultiplexer. Through numerical analyses using a 3-dimensional beam propagation method (3D-BPM, BeamPROP™, Keysight Technologies, Santa Rosa, CA, USA), the relative phase deviation (ΔΦ), defined as the phase difference from the bar port (Φ_Bar_) to the cross port (Φ_Cross_) of the 2:2 MMI coupler, was calculated.

Table 2 shows the summarized results on the three types of MMI couplers we used in this work. In Table 2, parameter L_π_ represents the distance at which the difference between the propagation constants of the fundamental mode and the first-order mode becomes π rad [12,13].

As the result of the 3D-BPM simulations, it was found to be +0.5π rad for an MMI coupler with a coupling ratio (κ_MMI_) of 0.5, −0.5π rad for κ_MMI_ = 0.85, and either −0.4π or −0.6π rad for κ_MMI_ = 0.72, depending on the input channel position. Unlike directional couplers (DCs), where ΔΦ remains a constant −0.5π rad regardless of the coupling ratio (κ_DC_), these discrete MMI phase shifts act as a hurdle to realizing the desired optical demultiplexing spectral response unless proper phase matching is applied at each MDI stage.

In this study, an MZI consisting of MMI couplers was designed and evaluated to more clearly observe the MMI coupler’s splitting and phase responses compared to characterizing a single MMI component. For instance, when evaluating MMI couplers with κ_MMI_ = 0.85 and κ_MMI_ = 0.72, clearly analyzing the transmission spectra is challenging due to their relatively similar splitting ratios. By constructing MZIs based on MMI couplers, the transmittance differences between the bar and cross ports become more pronounced than in a single-component setup. This is driven by amplitude and phase interactions within each MMI region, allowing for a more accurate analysis of the MMI splitting properties in the O-band regime.

**Figure 1:**
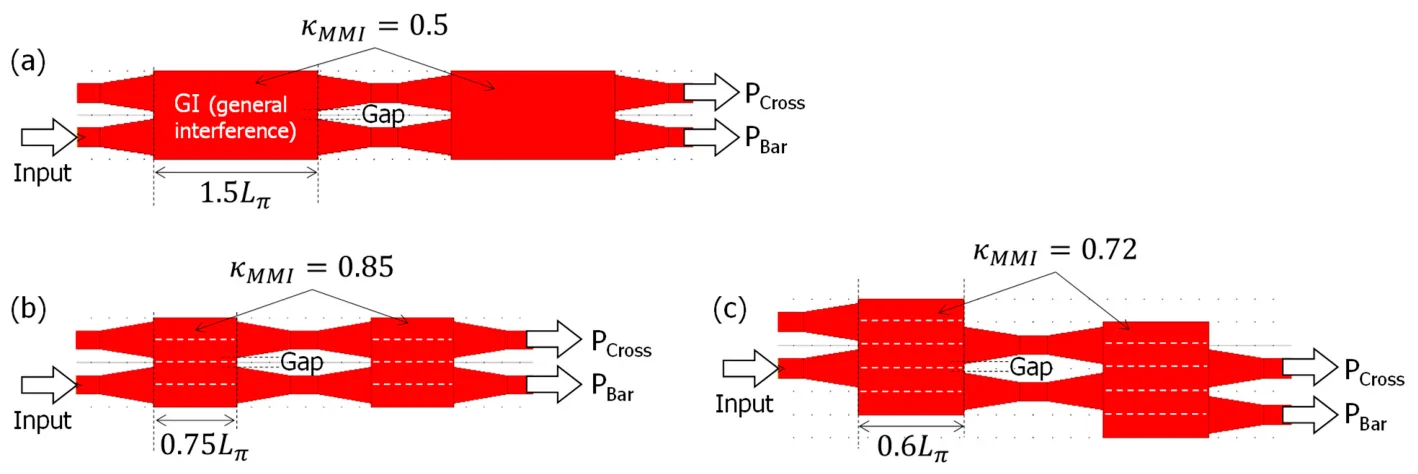
Schematic diagrams of silicon-nanowire-type MMI-based Mach–Zehnder interferometers (MZIs): (**a**) based on the 3 dB MMI couplerswith κ_MMI_ = 0.5, (**b**) based on the MMI couplers with κ_MMI_ = 0.85, and (**c**) based on the MMI couplers with κ_MMI_ = 0.72.

Figure 1 illustrates the MMI-based MZIs. The configurations consist of general interference (GI)-based MMI couplers with κ_MMI_ = 0.5 (a) [12], MMI couplers with κ_MMI_ = 0.85 (b) [13], and MMI couplers with κ_MMI_ = 0.72 (c) [13]. Notably, these schemes do not incorporate any optical delay lines in the MZI arms.

For the designs in Figure 1a,b, the MMI couplers exhibit axial symmetry, ensuring that the ΔΦ values remain constant regardless of the input port position. In contrast, the MMI coupler in Figure 1c possesses point symmetry, which causes ΔΦ to deviate by 0.2π rad depending on the input port location. The MMI-based MZIs were designed using RSoft Photonic Design Tools (CAD™, Keysight Technologies, Santa Rosa, CA, USA). Assuming a silicon-nanowire single-mode waveguide with a width of 0.41 µm and a height of 0.22 µm (FemSIM™, Keysight Technologies, Santa Rosa, CA, USA), all MMI sections were parameterized based on standard self-imaging principles [12,13]. Taper structures were implemented at each MMI region to control the excited modes. To facilitate fabrication and improve production yield, the gap widths for all MZI schemes were uniformly set to 0.2 µm. The waveguide parameters of the three types of MMI couplers were set in the following ways: the MMI width (W_MMI_) and length (L_MMI_) are designed as follows: W_MMI_ = 2.0 [μm], L_MMI_ = 18.4 [μm] for κ_MMI_ = 0.5, W_MMI_ = 2.0 [μm], L_MMI_ = 9.3 [μm] for κ_MMI_ = 0.85, and W_MMI_ = 2.5 [μm], L_MMI_ = 11.5 [μm] for κ_MMI_ = 0.72.

**Figure 2:**
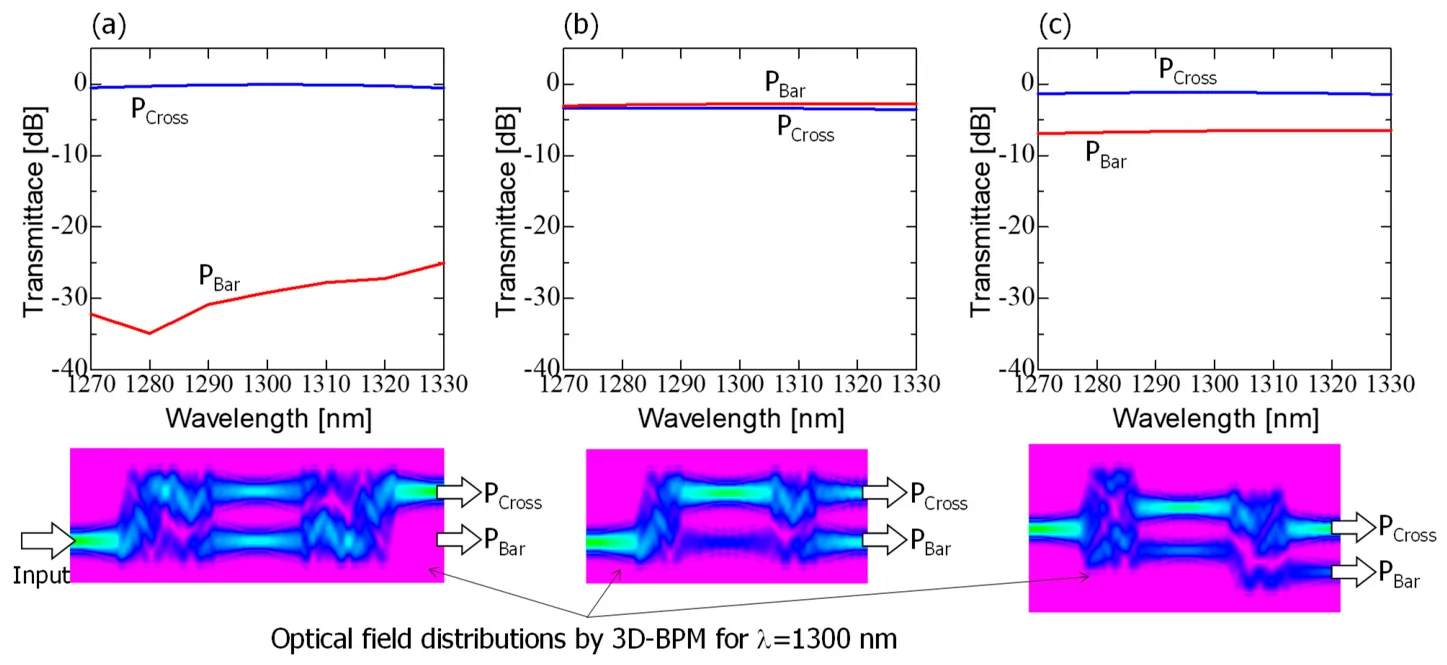
Calculated transmittances for the MMI-based MZIs: (**a**) based on the 3 dB MMI couplers with κ_MMI_ = 0.5, (**b**) based on the MMI couplers with κ_MMI_ = 0.85, and (**c**) based on the MMI couplers with κ_MMI_ = 0.72, together with optical field distributions by the 3D-BPM (BeamPROP^TM^, Keysight Technologies, Santa Rosa, CA, USA) at λ = 1300 nm.

Figure 2 illustrates the calculated optical transmittances and optical field distributions (at λ = 1300 nm) for the three types of MMI-based MZIs via the 3D-BPM approach (BeamPROP^TM^, Keysight). For all the MMI-based MZIs depicted in Figure 2, the input port was assigned to the lower side. As shown in Figure 2a, the optical fields are evenly split by the initial GI-based MMI. The two signals, propagating with a phase difference of ΔΦ = +0.5π rad in the MZI arms, predominantly couple to the output port P_Cross_. Due to the equal splitting intensity and the discrete phase deviation, almost no optical power couples to the P_Bar_ port. This trend is robustly maintained across the O-band spectral range. While the theoretical extinction ratio (ER) between the two output ports can exceed 40 dB, the practical ER may degrade depending on the degree of minor power imbalances.

In Figure 2b, the asymmetric splitting ratio of κ_MMI_ = 0.85 directly governs the optical transmittances at the two outputs. The asymmetrically split signals, possessing a phase difference of ΔΦ = −0.5π rad in the MZI arms, couple to the P_Bar_ and P_Cross_ ports at a nearly identical rate. It should be noted that although the ΔΦ values are completely out of phase between the cases in Figure 1a,b [+0.5π vs. −0.5π rad], the dominant factor determining the optical transmittance levels in these isolated MZI schemes is κ_MMI_. Therefore, this out-of-phase relationship has no significant influence on the individual transmittance levels. However, when an MZI is constructed by combining two different MMI couplers possessing an out-of-phase relationship, this phase mismatch strongly influences the overall optical transmission, as previously reported [16].

Similarly, in Figure 2c, the asymmetric splitting ratio of κ_MMI_ = 0.72 dictates the discrete and imbalanced transmittances for the two outputs. The asymmetrically split optical signals, carrying a phase difference of ΔΦ = −0.4π or −0.6π rad (depending on the input channel location), couple to the P_Cross_ and P_Bar_ ports with an imbalanced ratio of approximately 3.5:1. This corresponds to an ER of ~5.5 dB between the two output ports.

As seen in Figure 2a–c, the transmittances for each case are apparently different from each other, which allows us to more precisely estimate coupling behaviors. Overall, although it is fundamentally difficult to isolate the precise phase values, such as the exact sign of ΔΦ (i.e., distinguishing between +0.5π rad and −0.5π rad) or the 0.2π rad offset, purely from the intensity response of these specific test structures, this MZI-based configuration offers a significant advantage for accurately characterizing the MMI coupling ratios. Specifically, evaluating the precise splitting ratios (i.e., 50:50, 85:15, and 72:28) through MZI transmittance measurements provides much higher accuracy than directly measuring the characteristics of a single standalone MMI coupler. It should be noted that perfectly isolating the phase information would require adopting specific MZI schemes that combine different MMI types (e.g., κ_MMI_ = 0.5 and κ_MMI_ = 0.85 within the same MZI).

**Figure 3:**
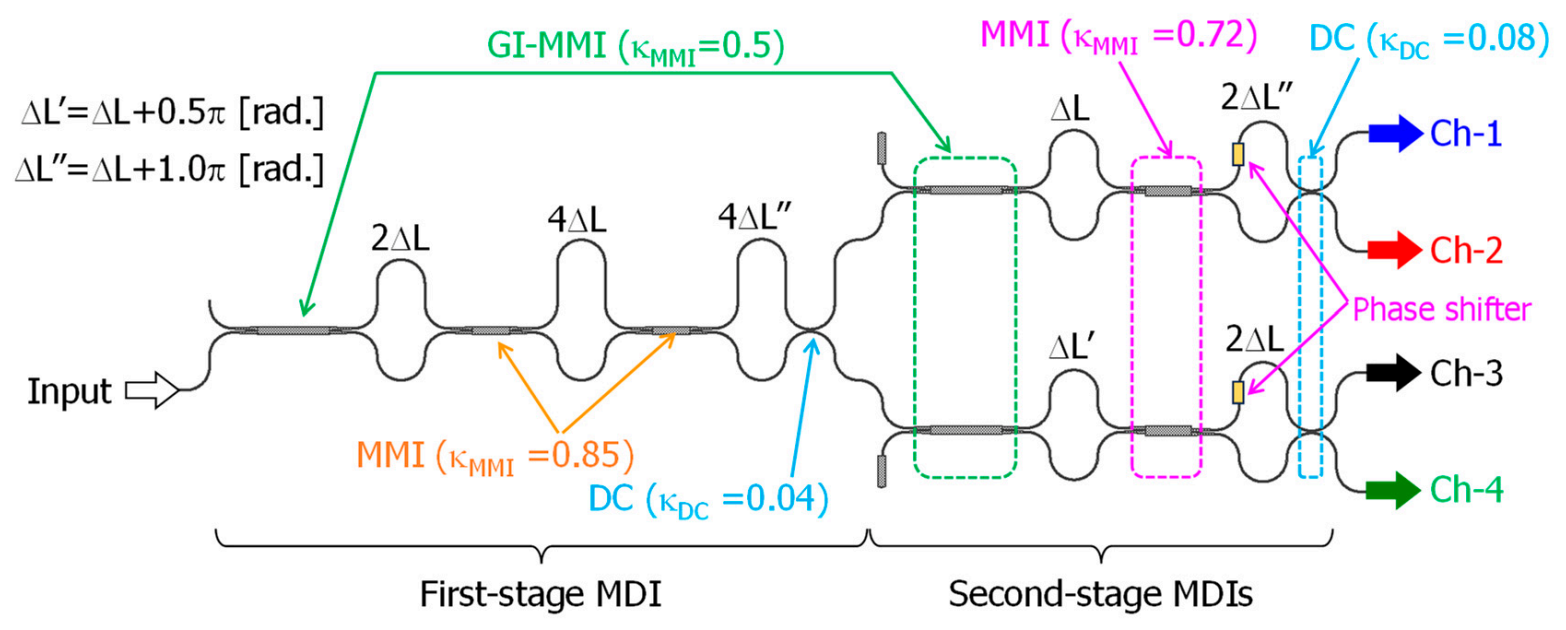
CWDM-targeted MMI-based MDI-type optical demultiplexer for flat-topped spectral response.

Figure 3 presents a schematic diagram of the MMI-based MDI-type CWDM optical demultiplexer. For handling 4-channel CWDM operations, the device comprises two MDI stages configured with pre-defined coupling coefficients (κ_MMI_ and κ_DC_) and optical path differences (integer multiples of ΔL combined with specific phase shifters) to synthesize flat-topped passbands.

The fundamental delay length ΔL was established to provide a 20 nm channel spacing in the O-band regime. In our previous work, we adopted a similar MMI-based MDI scheme but were unable to experimentally verify the spectra flat demultiplexing behavior due to the unclarified discrete phase responses of each MMI coupler. Specifically, the out-of-phase relationship between κ_MMI_ = 0.5 and κ_MMI_ = 0.85 in the first-stage MDI severely distorted the filtering response. Resolving this issue necessitates the introduction of additional phase shifters within each delay line to preserve an in-phase relationship for every CWDM channel grid.

Table 3 details the extra phase adjustments required at each delayed interferometer to maintain this in-phase condition. In the prior study, while the theoretical phase conditions were provided, satisfying them demanded excessively large phase variations in the first-stage MDI. As summarized in Table 3, the present work optimizes the phase parameters primarily in the second-stage MDIs without adding extra phase shifts to the first stage, thereby minimizing the overall required phase corrections. The waveguide width and bending radius for the device shown in Figure 3 were chosen as 350 nm and 6 µm, respectively, ensuring single-mode operation within the CWDM spectral regime.

The parameters for the three MMI types are identical to those used in Figure 1. Furthermore, in the second-stage MDIs (Figure 3), specific phase shifters can be inserted immediately after the κ_MMI_ = 0.72 couplers to enhance the spectral quality by reducing excess loss and out-of-band crosstalk. Table 4 outlines the applied phase variations for these shifters (Δϕ_PS_) and their corresponding second delay lines in the second-stage MDIs.

Case-A represents the baseline condition with no additional phase shifters (equivalent to Table 3). In Case-B, extra phase shift of −0.2π rad is added to each κ_MMI_ = 0.72 coupler, effectively reducing the total delay line lengths in the second-stage MDIs by 0.2π rad. Conversely, in Case-C, a phase shift of +0.2π rad is applied, increasing the total delay line length by +0.2π rad.

**Figure 4:**
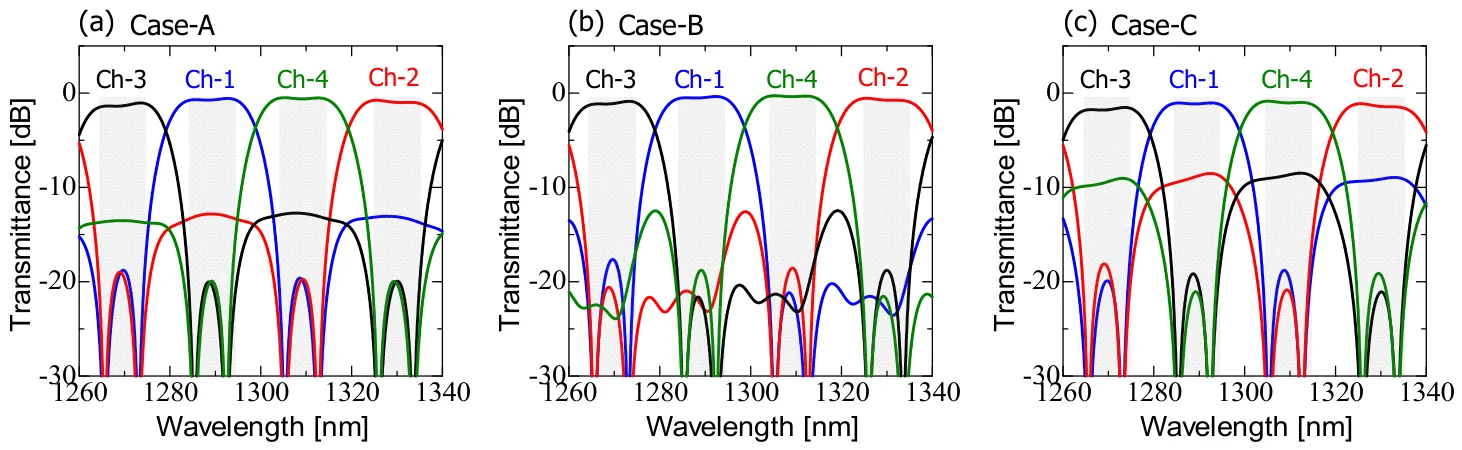
Analytically calculated spectral responses for the proposed device scheme shown in Figure 3: (**a**) Case-A, (**b**) Case-B and (**c**) Case-C.

Utilizing coupled-mode theory and the transfer matrix method, we analytically calculated the TE-mode transmission spectra of the proposed device under the conditions listed in Table 3 and Table 4. Figure 4 displays the analytical results for (a) Case-A, (b) Case-B, and (c) Case-C.

As shown in Figure 4a, implementing properly distributed MMI phase compensations successfully achieves flat-topped spectral responses across the entire O-band. However, in Case-A, the out-of-band spectral crosstalk from neighboring channels is limited to approximately −13 dB. This limitation arises primarily from the inherent phase mismatch between the MMI coupler (κ_MMI_ = 0.72) and the DC (κ_DC_ = 0.08), caused by the 0.2π-rad ΔΦ difference dependent on the input port position.

By introducing phase shifters with signs opposite to this ΔΦ discrepancy, the phase mismatch can be effectively compensated, further suppressing the spectral crosstalk to the −20 dB level. As illustrated in Figure 4b, the Case-B scheme demonstrates significantly improved low-crosstalk characteristics without sacrificing spectral flatness, excess loss, or available flatband range. In contrast, if the applied phase shifters share the same sign as the ΔΦ difference (Case-C), the phase mismatch between the MMI coupler and the DC is exacerbated, leading to a severe degradation in spectral quality, as evidenced in Figure 4c.

## 3. Fabrication and Experimental Characterizations

The MMI-based MZIs and MDI-type CWDM demultiplexers discussed in the previous section were fabricated using a standard silicon photonics foundry process based on 193 nm ArF-dry lithography, utilizing a 220 nm thick silicon core layer and a 2 µm thick buried oxide (BOX) layer. To characterize the transmission spectra, a tunable laser with a tuning range of 1270 nm to 1330 nm was employed as the optical source, and the spectral responses were evaluated using an optical vector analyzer (OVA, LUNA 5013, LUNA Technologies, Roanoke, VA, USA).

**Figure 5:**
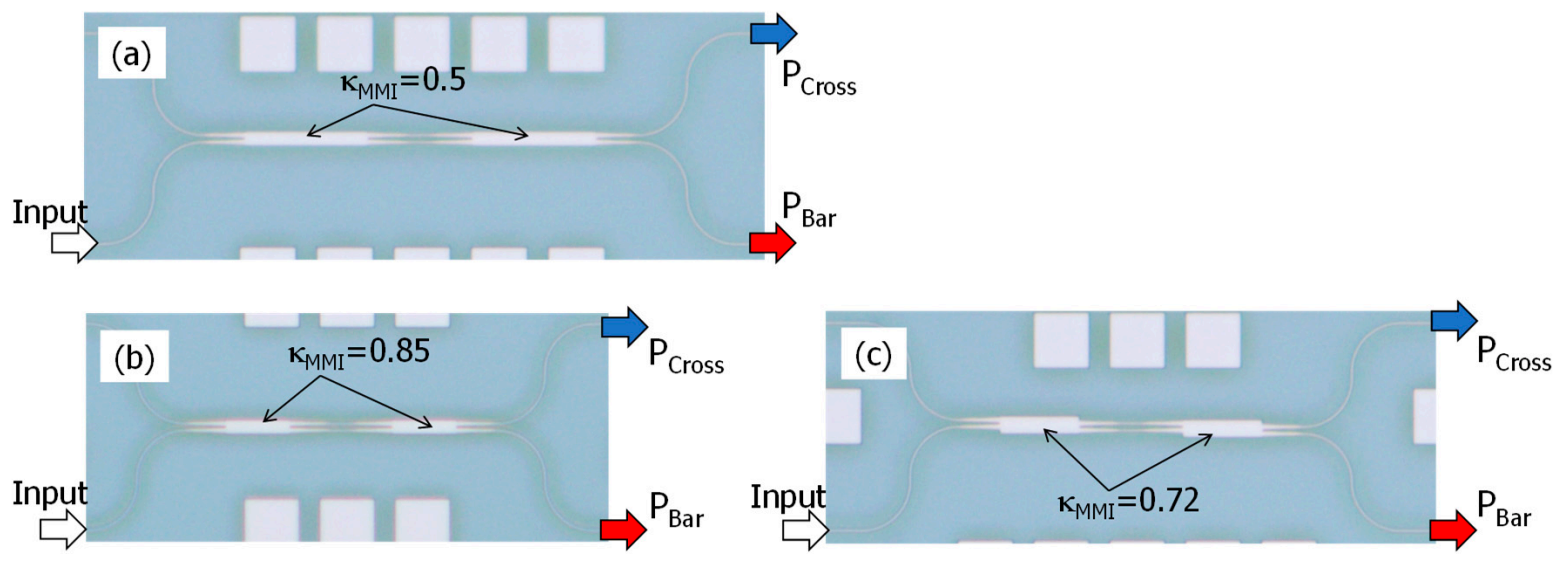
Fabricated silicon-nanowire-type MZIs: (**a**) based on 3 dB GI-based MMI couplers with κ_MMI_ = 0.5, (**b**) based on the MMI couplers with κ_MMI_ = 0.85, and (**c**) based on the MMI couplers with κ_MMI_ = 0.72.

Figure 5 displays microscopic images of the fabricated silicon-nanowire MZI configurations based on 3 dB GI-type MMI couplers with κ_MMI_ = 0.5 (a), MMI couplers with κ_MMI_ = 0.85 (b), and MMI couplers with κ_MMI_ = 0.72 (c). The geometrical parameters of the waveguides and the three MMI variants were identical to those described in Section 2. To facilitate coupling to single-mode fibers (SMFs) at the chip facets, the input and output ports were spatially separated by 30 µm using S-shaped bending regions.

**Figure 6:**
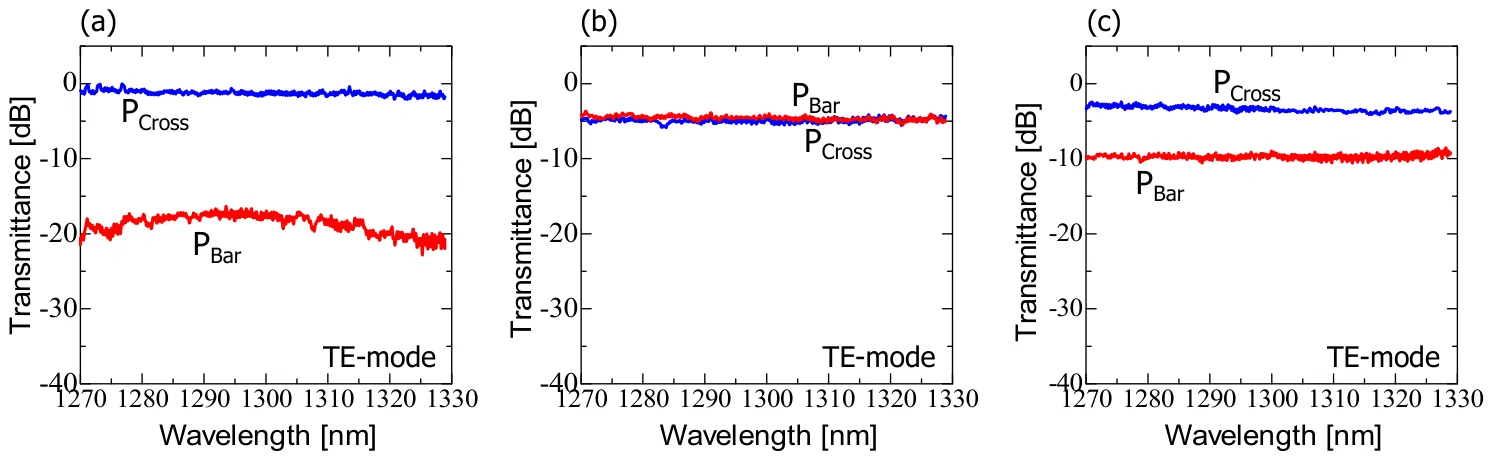
Measured transmission spectra (TE-mode) for the fabricated silicon-nanowire-type MZIs: (**a**) based on the 3 dB GI-based MMI couplers with κ_MMI_ = 0.5, (**b**) based on the MMI couplers with κ_MMI_ = 0.85, and (**c**) based on the MMI couplers with κ_MMI_ = 0.72.

Figure 6 presents the measured transmission spectra for the corresponding MZI structures. These spectra represent TE-mode input to TE-mode output configurations and intrinsically include the excess losses at the MMI regions, which were omitted in the idealized simulations, as well as wavelength-sensitive MMI losses. As observed in Figure 6a, the input signal predominantly couples to the P_Cross_ port rather than the P_Bar_ port. The measured ER between P_Cross_ and P_Bar_ is approximately 20 dB across the O-band spectral range. While the spectral behaviors align well with the theoretical predictions shown in Figure 2a, the measured ER of ~20 dB is lower than the simulated ideal value. This discrepancy is most likely due to a slight power imbalance induced by the initial 3 dB MMI coupler.

In Figure 6b, the asymmetric splitting ratio of κ_MMI_ = 0.85 results in nearly equal optical transmittances at both the P_Cross_ and P_Bar_ ports. This trend is consistently maintained across the O-band regime, exhibiting excellent agreement with the simulation results from Figure 2b. Conversely, the distinct asymmetric splitting ratio of κ_MMI_ = 0.72 induces discrete, imbalanced optical transmittances at the two outputs, as depicted in Figure 6c. Here, the measured ER between P_Cross_ and P_Bar_ is ~6 dB, which closely matches the simulated value of 5.5 dB derived from the discrete splitting ratio of 0.72 and the channel-dependent phase shifts (ΔΦ = −0.4π or −0.6π rad). Overall, the simulated and measured transmission characteristics demonstrate strong agreement, verifying the discrete amplitude splitting and phase changes governed by the MMI phenomena. The minor ER deviations between the calculations and measurements originate primarily from slight fabrication-induced κ_MMI_ mismatches. We deduce that the absolute magnitude of this κ_MMI_ mismatch is relatively uniform across the different MMI designs. However, the resulting impact on the ER varies due to the differing sensitivity of the MMI-based MZI schemes depending on the target κ_MMI_ value. As the optical splitting ratio approaches 0.5, the transmission sensitivity of the P_Bar_ port increases significantly. This explains why the experimental spectra for κ_MMI_ = 0.85 aligns more tightly with theoretical models [Figure 2b and Figure 6b] compared to the κ_MMI_ = 0.5 case [Figure 2a and Figure 6a], where the ideal ER is more severely degraded by minor process variations.

**Figure 7:**
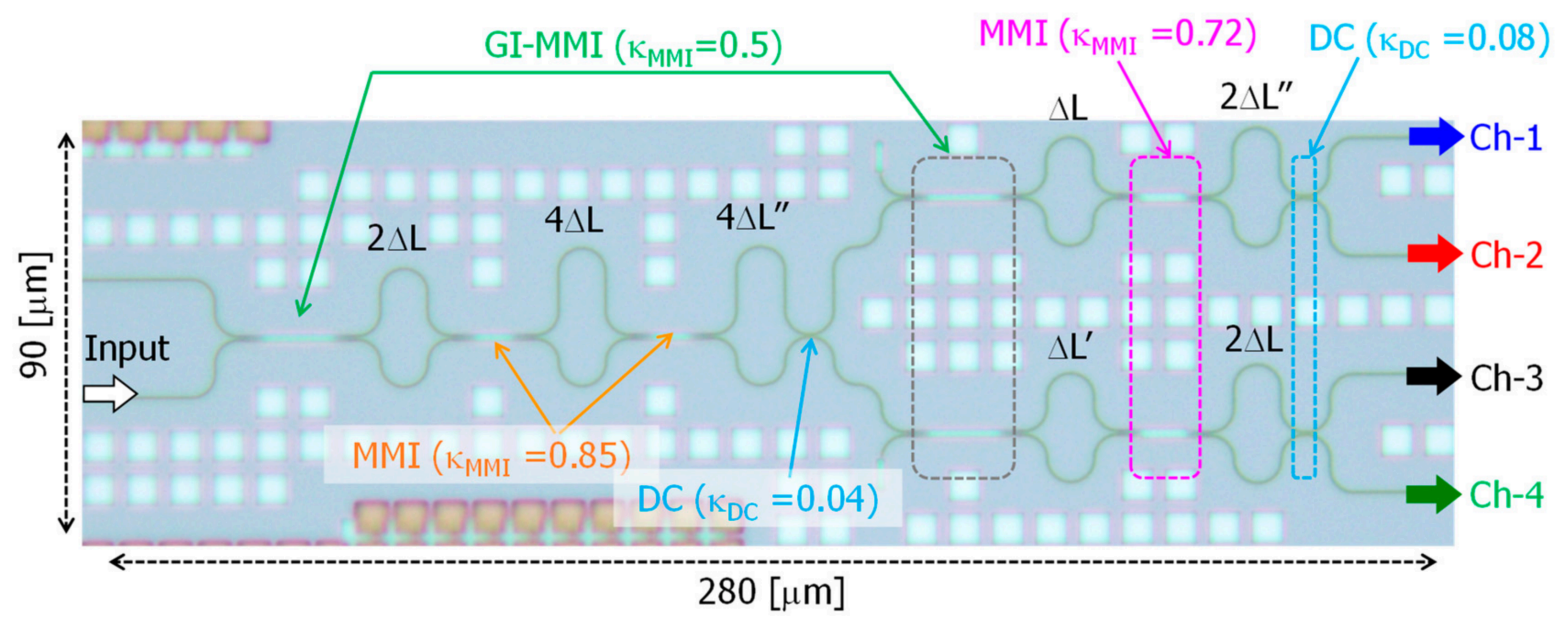
Fabricated CWDM-targeted MDI-based optical demultiplexers for box-like spectral response. The fabricated chip was designed as Case-A.

Considering the amplitude and phase responses of the verified MMI couplers, the complete MMI-based MDI-type CWDM optical demultiplexer was fabricated according to the schematic in Figure 3. Figure 7 shows a microscopic image of the fabricated device, designed specifically as Case-A, implying that no specific phase shifters were placed between the κ_MMI_ = 0.72 MMI couplers and the κ_DC_ = 0.08 DCs. The chip footprint is approximately 90 µm in width and 280 µm in length.

**Figure 8:**
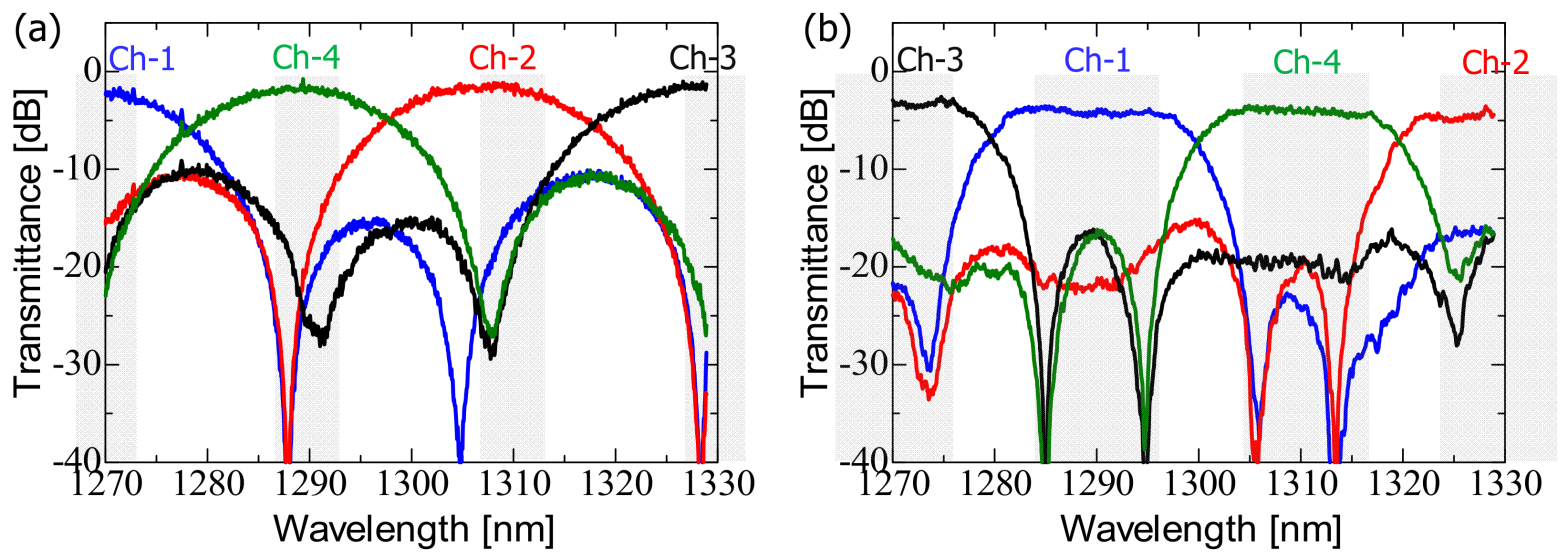
Measured transmission spectra of the fabricated MMI-based MDI-type CWDM demultiplexers designed for Gaussian filter spectral shape (**a**) and box-like filter spectral shape (**b**).

Using the aforementioned experimental setup, we evaluated the transmission spectral responses of the fabricated demultiplexers. Figure 8 compares the measured spectra for two MMI-based MDI-type devices. In Figure 8a, a simpler device comprising only three 3 dB GI-based MMI couplers designed to yield a Gaussian-shaped filter response was characterized. While a filtering response was achieved at each CWDM channel grid, the spectral profile assumes a Sinc-function shape rather than a box-like one. Note that the truncation of the spectral response at the shorter and longer wavelength extremes is solely due to the limited tunable range of the laser sources (1270 nm to 1330 nm).

In contrast, the proposed device depicted in Figure 7 was specifically engineered to achieve a flat-topped response through the cascaded MDI architecture. As shown in Figure 8b, clear box-like spectral responses were observed across all output channels. The 1 dB filtering bandwidth was measured to be >13.2 nm (reaching up to 13.9 nm) for Ch-1 and Ch-4, which is more than twice as wide as that of the Gaussian-shaped filter in Figure 8a. Regarding the 1 dB bandwidth of the outermost channels, it is fundamentally governed by the waveguide dispersion. In silicon nanowires, the modal confinement changes with wavelength, causing the flat bandwidth to slightly decrease at shorter wavelengths (i.e., 1270 nm) and increase at longer wavelengths (i.e., 1330 nm). Through our measurements, we confirmed that the middle channels exhibit a 1 dB bandwidth of >13.2 nm. Given this wide margin, we can theoretically and safely deduce that the 1270 nm channel still satisfies the >12 nm flat bandwidth condition despite the slight dispersion-induced narrowing.

By properly compensating for the out-of-phase relationships within each MDI stage as outlined in Table 3, we successfully realized a spectrally flat CWDM response without the spectral degradations observed in our prior work. The excess loss in Figure 8b is estimated at 4–5 dB within the O-band regime, which is approximately 2 dB higher than the device in Figure 8a due to the increased number of MMI couplers. Notably, the measured transmittance exhibits a slight downward trend at longer wavelengths, suggesting that the optimal operating wavelength center has shifted toward the shorter wavelength side. This is likely caused by an unintentional narrowing of the MMI widths during the fabrication process. Thus, with further dimensional optimization, the excess loss of the proposed device could be notably reduced by more than 2 dB.

**Figure 9:**
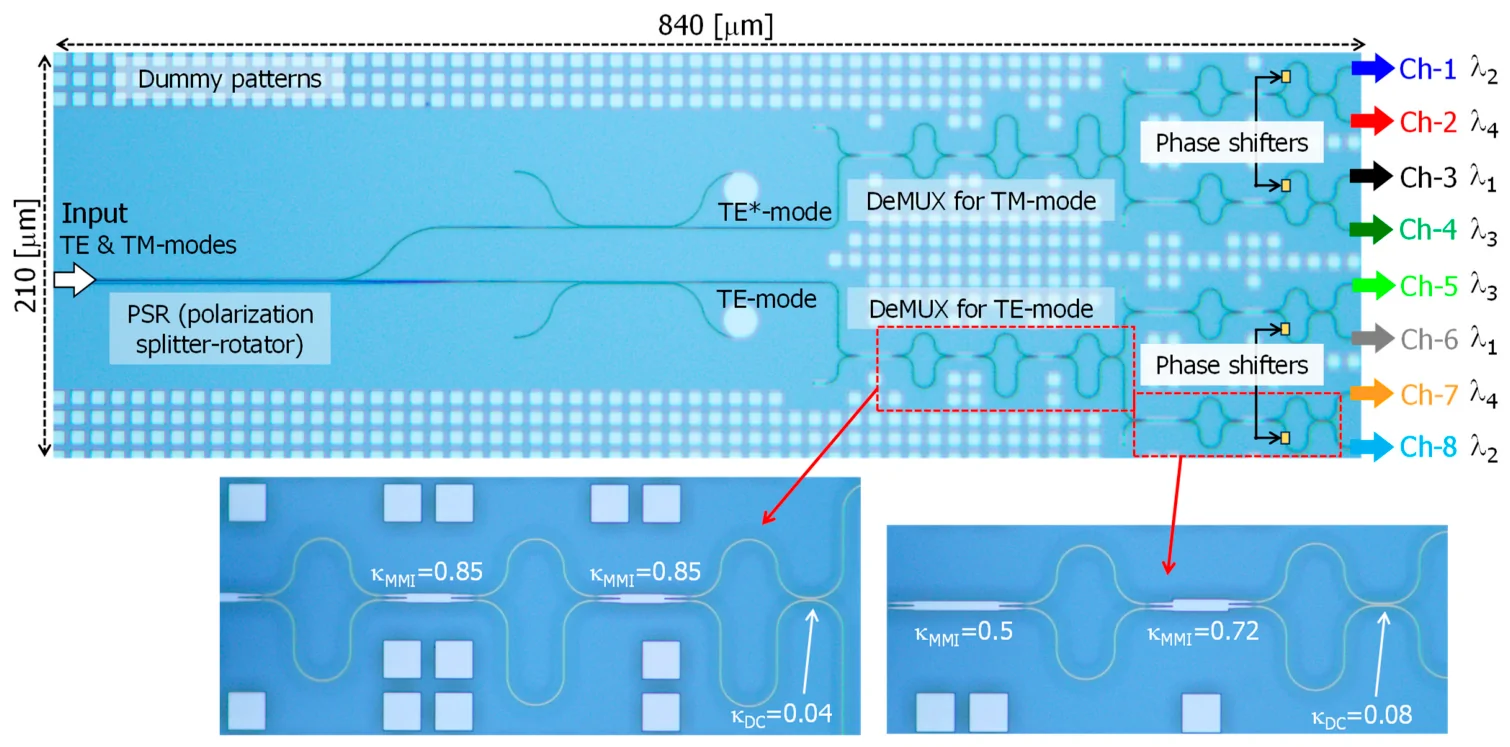
Fabricated polarization-diversified scheme with the polarization splitter-rotator (PSR) and MMI-based MDI-type CWDM optical demultiplexers, together with magnified views around the lower-side demultiplexer area. TE*-mode represents the polarization state controlled from the initial TM-mode. Three types of devices (Case-A, Case-B and Case-C) were fabricated on the same die.

For practical deployment in optical receivers, demultiplexers must operate reliably under arbitrary states of polarization due to the random nature of signals propagating through SMFs. While the CWDM demultiplexer shown in Figure 7 functioned properly for TE-polarized light, silicon-nanowire waveguides and coupler schemes inherently exhibit strong structural birefringence, typically restricting their use to TE-mode inputs. To overcome this limitation, we integrated a polarization-handling device into the demultiplexer architecture to accommodate arbitrary input polarization states through a polarization diversity configuration [14,15,19]. Figure 9 displays the fabricated polarization-diversified scheme, combining a polarization splitter-rotator (PSR) with the two identical MMI-based MDI-type CWDM demultiplexers. The MDI demultiplexers positioned centro-symmetrically in Figure 9 replicate the structures described in Table 3 and Table 4, with three variations (Case-A, Case-B, and Case-C) fabricated on the same die. Because the specific phase shifters operate by adjusting the waveguide length, there is no need for active refractive index control or additional fabrication steps. The total footprint of this integrated chip is approximately 840 µm in length and 210 µm in width.

Within the PSR region, a randomly polarized input signal is initially separated into TM- and TE-modes by a DC-based splitter. Subsequently, the TM-mode component is rotated into the TE-mode utilizing a partially etched ridge structure. Consequently, the PSR outputs two TE-mode signals, regardless of the initial input state, are independently processed by the identically designed TE-mode demultiplexers. In a fully packaged optical receiver, these co-polarized output signals can be processed to and electrically terminated at a monolithic Ge-on-Si photodetector equipped with dual-input waveguides [20,21]. To independently verify its performance of polarization handling properties, an isolated PSR was also characterized, exhibiting an excess loss of <2 dB and a polarization-dependent loss (PDL) of <1 dB across the measured spectral range.

**Figure 10:**
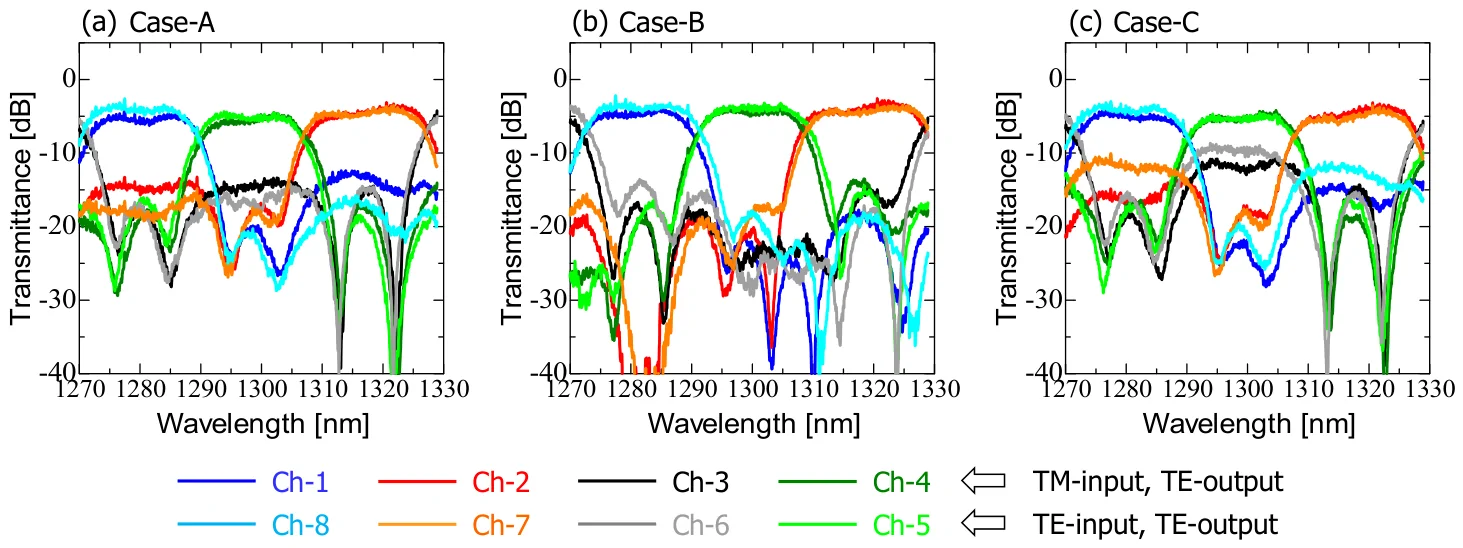
Measured transmission spectra of the fabricated PSR-integrated optical demultiplexer (shown in Figure 9) of TM-TE spectra for the output port Ch-1 to Ch-4 and TE-TE spectra for the output port Ch-5 to Ch-8: (**a**) Case-A, (**b**) Case-B, and (**c**) Case-C.

Figure 10 presents the measured transmission spectra of the fabricated PSR-integrated optical demultiplexers corresponding to (a) Case-A, (b) Case-B, and (c) Case-C. In these plots, output ports Ch-1 to Ch-4 represent the TM-input to TE-output responses, whereas Ch-5 to Ch-8 indicate the TE-input to TE-output responses. The devices exhibited almost identical flat-topped spectra irrespective of the phase control condition applied between the κ_MMI_ = 0.72 MMI coupler and the κ_DC_ = 0.08 DC. However, the overall spectral quality varied significantly depending on this phase condition. Crucially, all fabricated devices demonstrated polarization-insensitive transmission efficiencies, confirming that the initial TM-mode inputs were stably rotated and processed by the integrated PSR.

Comparing Figure 10a,b, the spectral qualities of Case-B are demonstrably superior to those of Case-A in terms of out-of-band crosstalk and excess loss. This improvement is directly attributed to the specific −0.2π-rad phase shifters, which effectively compensated for the inherent phase mismatch. Conversely, applying a phase shift with the opposite sign (Case-C) further degraded the spectral quality, as evidenced in Figure 10c and corroborating the theoretical predictions from Figure 4.

For the optimized Case-B device, we achieved an excess loss of <4.6 dB and a PDL of <1 dB, inclusive of the PSR across the entire measured spectral range. Although this device incorporates an additional PSR component, its total excess loss is comparable to that of the standalone MDI demultiplexer [Figure 8b]. This was achieved by slightly reducing the MMI length to pre-compensate for the fabrication-induced blue-shift observed earlier. Note that while the four channel grids are spaced by approximately 20 nm, the absolute filtering centers shifted to 1270, 1290, 1310, and 1330 nm due to effective refractive index variations in the waveguides. This offset can be easily corrected in future iterations by fine-tuning the waveguide widths or incorporating localized phase control regions.

In this work, spectral crosstalk from neighboring channels originates from the imperfect optical interference within the MDI architecture itself. It represents the signal leakage between neighboring channels propagating with the same polarization state. Here, the amount of spectral crosstalk was estimated by taking the difference in the primary port transmission rate from the sum of the transmission rates of all remaining output ports. Table 5 summarizes the measured 1 dB bandwidth and estimated spectral crosstalk for the measured output ports (six ports within 1270 to 1330 nm) shown in Figure 10b. As seen in Table 5, the flat bandwidth for each output channel was measured to be wider than 12 nm, which makes it possible to endure a wide range of temperature change or laser oscillation wavelength deviation. As for the spectral crosstalk suppression from other neighboring channels, its estimated value ranges from 21.1 dB (best case) to 7.7 dB (worst case).

**Figure 11:**
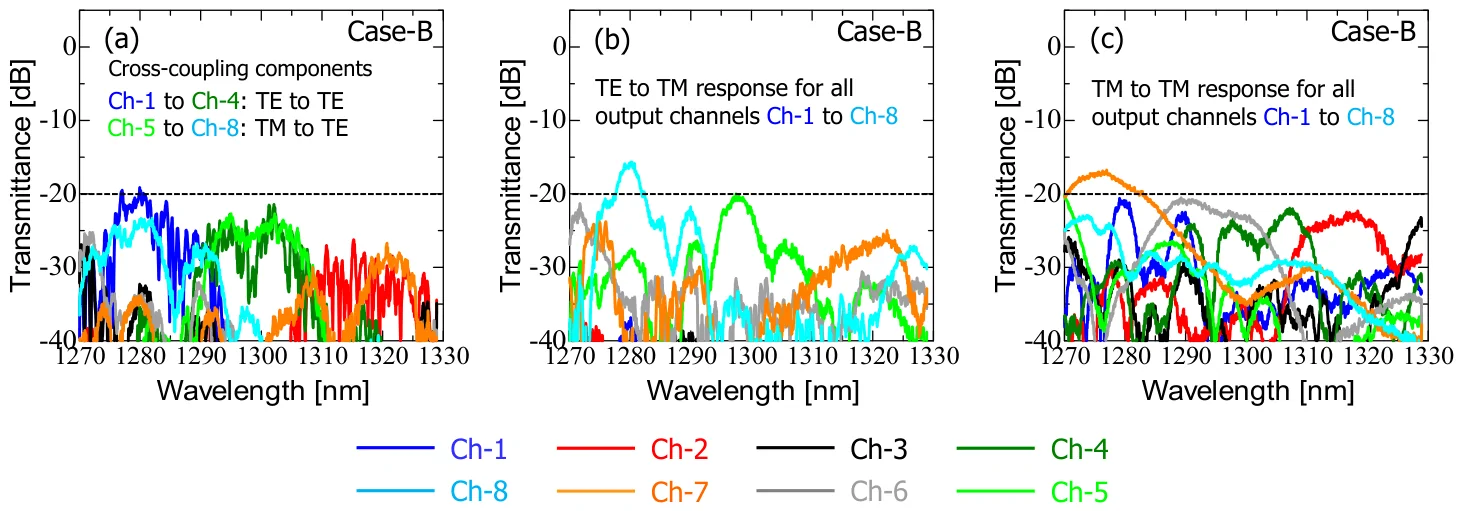
Measured polarization crosstalk responses of the Case-B device: (**a**) TE-TE responses for Ch-1 to Ch-4 and TM-TE responses for Ch-5 to Ch-8, (**b**) TE-TM responses for all output channels, and (**c**) TM-TM responses for all output channels. Each case for spectral relations of arbitrary-to-arbitrary polarization signals was evaluated by using the optical vector analyzer (OVA, LUNA 5013).

In addition to the primary signal responses, we experimentally evaluated various polarization crosstalk components for the Case-B device using the OVA, as depicted in Figure 11. Polarization crosstalk strictly originates from the structural imperfections of the integrated PSR, leading to unwanted cross-coupling between TE- and TM-modes. In a sense, these components represent unwanted noise that must be minimized in practical systems. The measured spectra include (a) cross-coupled noise components (TE-TE for Ch-1 to Ch-4 and TM-TE for Ch-5 to Ch-8), (b) TE-TM responses for all output channels, and (c) TM-TM responses for all output channels.

We confirmed remarkably low polarization crosstalk levels below −20 dB for all measured polarization crosstalk components. Notably, the TM-TM response, which theoretically could exceed −20 dB in the shorter-wavelength region of the O-band due to insufficient handling ability of the single PSR, was also successfully maintained below −20 dB. This excellent TM-mode rejection is primarily attributed to the strong inherent birefringence of the silicon-nanowire waveguides constituting the MMI-based MDI demultiplexer.

Finally, for practical WDM applications, out-of-band spectral crosstalk from neighboring channels should ideally be minimized. While we theoretically identified that compensating the inherent phase offset at the κ_MMI_ = 0.72 coupler can reduce crosstalk to the −20 dB level [Figure 4b], the experimental crosstalk suppression [Figure 10b] was less pronounced than analytically predicted. To understand this discrepancy, we theoretically investigated the impact of random phase errors induced by fabrication imperfections. A uniform variation in waveguide width across a die primarily causes a global effective index change, which shifts the absolute center wavelengths of the WDM grid, rather than deteriorating the spectral shape itself. The spectral crosstalk degradation is fundamentally driven by line-edge roughness (LER) induced during the dry etching process. LER creates irregular, non-uniform fluctuations on the waveguide sidewalls along the propagation direction. As the optical signal travels through the long interferometric delay lines, these random physical fluctuations accumulate as statistical random phase errors, directly degrading the interference extinction and raising the crosstalk floor. Usually, the standard deviation of the random phase errors [ $\sigma \left(\delta \phi \right)$] for the Δ*L* delay line can be given by the following relation [22].

(1) $$\sigma \left(\delta \phi \right)=k_{0}\cdot \sigma \left(\delta w\right)\cdot \frac{dn_{eq}}{dw}\cdot \sqrt{∆L\cdot L_{c}}$$

where $k_{0}$, $\sigma \left(\delta w\right)$, $\frac{dn_{eq}}{dw}$, ∆L, and $L_{c}$ represent the propagation constant in free space, standard deviation of the LER, the variation rate of the equivalent refractive index against waveguide width, the path-length difference at MDI and the correlation length related to spatial frequency of the line width roughness, respectively.

**Figure 12:**
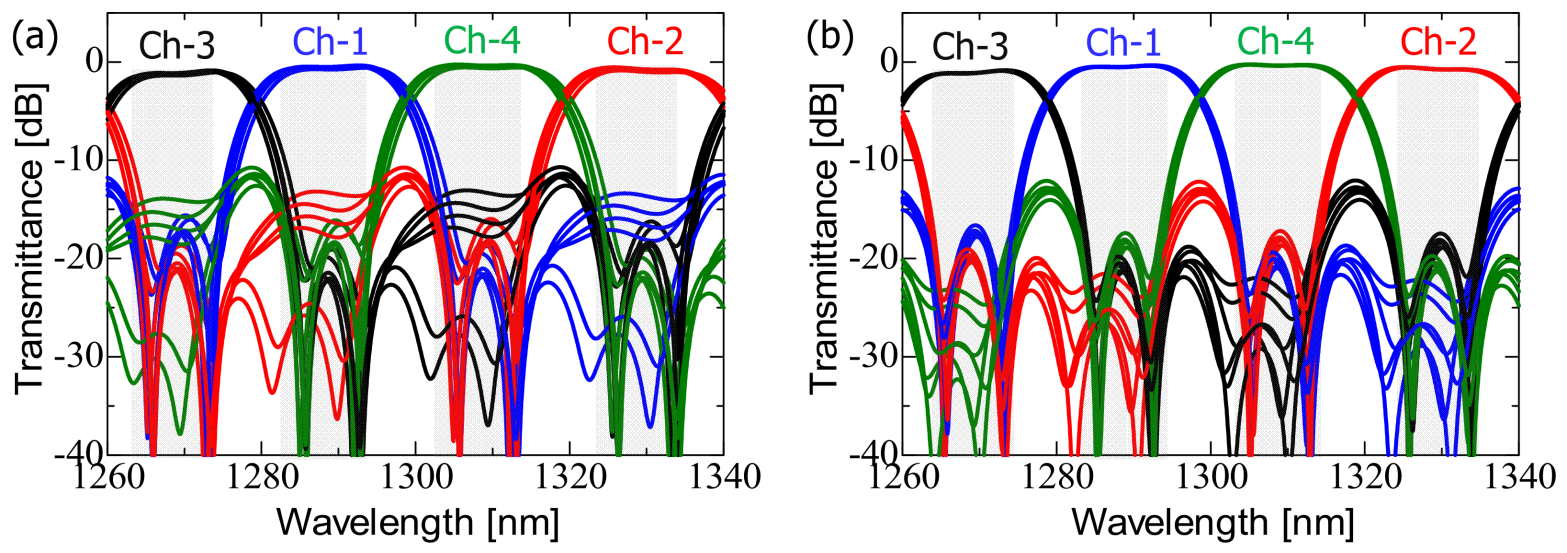
Analytically calculated transmission spectra of the MDI-type device (Case-B) when the random phase errors at each delay line in all MDI stages were generated by fabrication imperfections: the value of σ(δϕ) is set at 0.1π rad (**a**) and 0.04π rad (**b**).

Figure 12 illustrates the analytically calculated transmission spectra for the Case-B device when random phase fluctuations are introduced at each delay line in all MDI stages. Based on normal statistical distribution of the phase fluctuations, the same simulations were iterated six times. In Figure 12, each case represents the spectra for the value of σ(δϕ) set at 0.1π rad (a) and 0.04π rad (b). As shown in Figure 12a, such unintended phase variations deteriorate the spectral crosstalk up to −12 dB. These spectral trends are close to our experimentally observed spectra shown in Figure 10b. Meanwhile, when it comes to reducing the value of σ(δϕ) to 0.04π rad as seen in Figure 12b, spectral crosstalk level can be suppressed to −18 dB. For instance, broadening the waveguide widths in the interferometric delay sections can significantly lower the value of $dn_{eq}/dw$, minimizing random phase fluctuations caused by LER [23]. Additionally, employing more precise fabrication technologies, such as ArF-immersion lithography [24], or incorporating a double-filtering scheme [5,7,18] into the proposed architecture would effectively suppress the out-of-band spectral crosstalk.

Overall, the proposed MMI-based CWDM optical demultiplexer provides excellent fabrication tolerance compared to pure DC-based devices, demonstrating a robust >12 nm 1 dB flat bandwidth, polarization insensitivity, and excellent polarization crosstalk suppression across the O-band regime.

## 4. Conclusions

In this study, we analytically designed and experimentally demonstrated CWDM-targeted, flat-topped optical demultiplexers based on distributed MMI phase compensations. The proposed architecture employs two-stage MDIs configured with discrete path-length differences and strategically placed phase shifters to synthesize spectrally flat, box-like CWDM filtering windows. The discrete coupling behaviors of three types of MMI couplers were identified both numerically and experimentally using MMI-based MZI structures whose intensity responses are more accurate than standalone MMI couplers.

The demultiplexers were fabricated using a commercial silicon photonics foundry platform based on 193 nm ArF-dry lithography. The initial baseline device was optimized to achieve a flatband, box-like filter response aligned with the CWDM channel grids, exhibiting excellent spectral flatness with 1 dB bandwidths exceeding 12 nm. This performance was enabled by strict phase matching at each MDI stage through proper phase adjustments tailored to the specific MMI phase behaviors.

Furthermore, to accommodate practical optical receiver applications, a polarization-diversified demultiplexer incorporating a monolithic PSR was developed. The fabricated device successfully demonstrated identical flat-topped CWDM responses (with a 1 dB bandwidth of >12 nm) regardless of the polarization state of the input signal. We experimentally validated that compensating for the inherent phase offset at the κ_MMI_ = 0.72 MMI coupler is critical for suppressing out-of-band spectral crosstalk. All measured polarization crosstalk components were robustly maintained below −20 dB across the O-band spectral regime. Although the current spectral crosstalk is ~−10 dB due to fabrication-induced random phase fluctuations, it can be further suppressed to −20 dB level through rigorous process optimization such as waveguide broadening or by adopting a double-filtering architecture. Consequently, this distributed MMI phase compensation strategy offers a highly practical and scalable solution for next-generation WDM datacom receivers.

## Figures and Tables

**Table 1:** Quantitative comparison of our recent studies on MDI-based optical demultiplexers.

	Refs. [14,15]	Ref. [16]	Ref. [17]	Ref. [18]
Filter spectral shape	Parabolic	Box-like (theory only)	Box-like	Box-like
Phase consideration btw 3-types of MMI couplers	NA	Not experimentally identified	Experimentally validated	Experimentally validated
Optimization of 72:28 MMI phase offset	NA	Theory only	NA	NA
Polarization handling	Pol. Diversified	NA	NA	Pol. diversified
WDM application areas	CWDM	CWDM	LR-8	CWDM

**Table 2:** General characteristics and relative phase relations for three types of MMI couplers.

	MMI Coupler 1	MMI Coupler 2	MMI Coupler 3
Coupling coefficient (k_MMI_)	0.5	0.85	0.72
Self-imaging principle	General interference	Restricted interference	Restricted interference
MMI region symmetry	Axial symmetric	Axial symmetric	Point symmetric
Interaction length for desired coupling operation	1.5L_p_	0.75L_p_	0.6L_p_
Relative phase difference (ΔΦ) [=Φ_Bar_ − Φ_Cross_]	+0.5π rad.	−0.5π rad.	−0.4π rad. or −0.6π rad. depending on input

**Table 3:** Parameters at the first-stage MDI and second-stage MDI schemes required for minimizing phase mismatch caused by the discrete MMI phase behavior for the three types of MMI couplers.

		Optimized Condition of Phase Shift Amount at Each Delay Line (This Work)	Previous Condition of Phase Shift Amount at Each Delay Line [16]
First-stage MDI	1st delay line	2ΔL	2ΔL − π rad.
	2nd delay line	4ΔL	4ΔL − 2π rad.
	3rd delay line	4ΔL + π rad.	4ΔL − π rad.
Second-stage MDIs	1st delay line for upper side	ΔL	ΔL + 0.5π rad.
	2nd delay line for upper side	2ΔL + π rad.	2ΔL
	1st delay line for lower side	ΔL + 0.5π rad.	ΔL
	2nd delay line for lower side	2ΔL	2ΔL + π rad.

**Table 4:** Conditions for the phase shift amount for each phase shifter and its corresponding 2nd delay line in the second-stage MDIs.

	Amount of the Phase Variation at Each Phase Shifter (Δϕ_PS_)	2nd Delay Line for Upper Side in the Second-Stage MDI	2nd Delay Line for Lower Side in the Second-Stage MDI
Case-A	0	2ΔL + π rad.	2ΔL
Case-B	−0.2π rad.	2ΔL + 0.8π rad.	2ΔL − 0.2π rad.
Case-C	+0.2π rad.	2ΔL + 1.2π rad.	2ΔL + 0.2π rad.

**Table 5:** Estimated 1 dB bandwidth and spectral crosstalk for the output ports from Figure 10b.

Output Port	1 dB Bandwidth	Spectral Crosstalk Suppression Ratio from Other Neighboring Channels	
		Best Value	Worst Value
Ch-1 (for TM-mode)	~13.5 nm	14.5 dB	8.8 dB
Ch-8 (for TE-mode)	~12.8 nm	21.1 dB	11.2 dB
Ch-2 (for TM-mode)	~13.7 nm	14.8 dB	10.6 dB
Ch-7 (for TE-mode)	~13.3 nm	18.9 dB	12.6 dB
Ch-3 (for TM-mode)	~14.4 nm	12.8 dB	7.7 dB
Ch-6 (for TE-mode)	~14.4 nm	19.2 dB	8.4 dB

## Data Availability

Data underlying the results presented in this paper are not publicly available at the time of publication, which may be obtained from the authors upon reasonable request.

## References

[1] Moralis-Pegios M., Pitris S., Alexoudi T., Terzenidis N., Ramon H., Lambrecht J., Bauwelinck J., Yin X., Ban Y., de Heyn P. (2020). 4-channel 200 Gb/s WDM O-band silicon photonic transceiver sub-assembly. Opt. Express.

[2] Asakawa K., Sugimoto Y., Nakamura S. (2020). Silicon photonics for telecom and data-com applications. Opto Electron. Adv..

[3] Hamdi M.M., Audah L., Rashid S.A., Al-Mashhadani M.A. (2020). Coarse WDM in metropolitan networks: Challenges, standards, applications, and future role. J. Phys. Conf. Ser..

[4] Horst F., Green W.M.J., Assefa S., Shank S.M., Vlasov Y.A., Offrein B.J. (2013). Cascaded Mach–Zehnder wavelength filters in silicon photonics for low loss and flat passband WDM (de-)multiplexing. Opt. Express.

[5] Dwivedi S., de Heyn P., Absil P., van Campenhout J., Bogaerts W. Coarse wavelength division multiplexer on silicon-on-insulator for 100 GbE. Proceedings of the 2015 IEEE 12th International Conference on Group IV Photonics (GFP).

[6] Xu H., Shi Y. (2018). Flat-top CWDM (De)Multiplexer based on MZI with bent directional couplers. IEEE Photon. Technol. Lett..

[7] Yen T.H., Hung Y.J. (2021). Fabrication tolerant CWDM (de)multiplexer based on cascaded Mach-Zehnder interferometers on silicon-on-insulator. J. Light. Technol..

[8] Yu L., Guo J., Xiang H., Yang G., Zhao Y., Li Y., Daoxin D. (2024). Ultra-compact and high-performance four-channel coarse wavelength-division (de)multiplexing filters based on cascaded Mach-Zehnder interferometers with Bezier-shape directional couplers. Opt. Express.

[9] Xu H., Lie L., Shi Y. (2018). Polarization-insensitive four-channel coarse wavelength-division (de)multiplexer based on Mach–Zehnder interferometers with bent directional couplers and polarization rotators. Opt. Lett..

[10] Siew S.Y., Li B., Gao F., Zheng H.Y., Zhang W., Guo P., Xie S.W., Song A., Dong B., Luo L.W. (2021). Review of silicon photonics technology and platform development. J. Light. Technol..

[11] Hammond A.M., Oskooi A., Chen M., Lin Z., Johnson S.G., Ralph S.E. (2022). High-performance hybrid time/frequency-domain topology optimization for large-scale photonics inverse design. Opt. Express.

[12] Soldano L.B., Pennings E.C.M. (1995). Optical multi-mode interference devices based on self-imaging: Principles and applications. J. Light. Technol..

[13] Bachmann M., Besse P.A., Melchior H. (1995). Overlapping-image multimode interference couplers with a reduced number of self-images for uniform and nonuniform power splitting. Appl. Opt..

[14] Jeong S.-H., Park P., Lee J.K. (2023). Polarization insensitive CWDM optical demultiplexer based on polarization splitter-rotator and delayed interferometric optical filter. Curr. Opt. Photonics.

[15] Jeong S.H., Park P., Lee J.K. (2023). Silicon-nanowire-type polarization-diversified CWDM demultiplexer for low polarization crosstalk. Nanomaterials.

[16] Jeong S.-H., Park P., Lee J.K. (2024). Experimental verification of phase behaviors of MMI couplers and their application to spectrally flat WDM (de)multiplexers. Appl. Opt..

[17] Jeong S.-H., Park P., Lee J.K. (2026). LR-8 optical demultiplexers based on silicon nanowires targeted for 400 GbE in datacenter applications. Appl. Opt..

[18] Jeong S.-H., Park P., Lee J.K. (2026). CWDM polarization diversified silicon-wire optical demultiplexers with spectral flatness and low crosstalk in O-band regime. J. Opt. Soc. Am. B.

[19] Sacher W.D., Barwicz T., Taylor B.J.F., Poon J.K.S. (2014). Polarization rotator-splitters in standard active silicon photonics platforms. Opt. Express.

[20] Dong P., Chen Y.-K., Buhl L. Reconfigurable four-channel polarization diversity silicon photonic WDM receiver. Proceedings of the Optical Fiber Communication Conferences (OFC 2015).

[21] Chen G., Yu Y., Zhang X. (2016). A dual-detector optical receiver for PDM signals detection. Sci. Rep..

[22] Goh T., Suzuki S., Sugita A. (1997). Estimation of waveguide phase error in silica-based waveguides. J. Light. Technol..

[23] Xu H., Dai D., Shi Y. (2021). Low-crosstalk and fabrication-tolerant four-channel CWDM filter based on dispersion-engineered Mach-Zehnder interferometers. Opt. Express.

[24] Horikawa T., Shimura D., Okayama H., Jeong S.-H., Takahashi H., Ushida J., Sobu Y., Shiina A., Tokushima M., Kinoshita K. (2018). A 300-mm silicon photonics platform for large-scale device integration. IEEE J. Sel. Top. Quantum Electron..

